# Supplementary material for: The Expression of Decidual Protein Induced by Progesterone (DEPP) Is Controlled by Three Distal Consensus Hypoxia Responsive Element (HRE) in Hypoxic Retinal Epithelial Cells
Source: Genes (Basel). 2020 Jan 18;11(1):111. doi: 10.3390/genes11010111 (PMC7016973; doi:10.3390/genes11010111)
Supplement: Supplementary file 1 [file genes-11-00111-s001.pdf]

Supplemental Material

Table S1. Genomic coordinates of HREs relative to the TSS

HRE	Sequence	Distance from TSS
1 [1)]	CCAGGTCC	- 350 / - 357
2	GTGTG**TGC**	- 367 / - 374
3	GTGTG**TGC**	- 823 / - 830
4 + 5 [2)]	GC**ACGTGC**	- 846 / - 853
6	AGACA**TGC**	- 883 / - 890
7	**GCA**CCTGG	- 1091 / - 1098
8	AC**ACG**CCT	- 1245 / - 1252
9	**GCA**GGTGG	- 1262 / - 1269
10	CCATG**TGC**	- 1393 / - 1400
11	**GCA**CATGG	- 1452 / - 1459
12	**GCA**CTTCT	- 1459 / - 1466
13	**GCA**CACAC	- 1670 / - 1677
14	**GCA**TGTCTG	- 1691 / - 1699
15	**GCA**GGTCC	- 1759 / - 1766
16	CCACC**TGC**	- 1778 / - 1785
17	**GCA**CCTCC	- 1838 / - 1845
18 [1)]	GGAAGTGG	- 2523 / - 2530
19	**GCA**AGTAT	- 2838 / - 2845
20	GTGCGTGC	- 2998 / - 3005
21 [1)]	GGACATGG	- 3092 / - 3099
22	**GCA**AGTGG	- 3191 / - 3198
23 [1)]	GGACCTGG	- 3314 / - 3321
24	CCAGG**TGC**	- 3339 / - 3346
<u>25</u>	<u>GGACG**TGC**</u>	<u>- 3425 / - 3432</u>
<u>26</u>	<u>**GCA**CATCT</u>	<u>- 3517 / - 3524</u>
<u>27</u>	<u>AC**ACG**CCC</u>	<u>- 3551 / - 3558</u>
28	**GCA**CGTAG	- 3563 / - 3570
29	GGATG**TGC**	- 3615 / - 3622

Bold characters indicate the conserved core of the HRE. Underlined HREs were identified as necessary to induce DEPP under hypoxic conditions. [1)] Identified by JASPAR database as HRE but lacking the CGT (sense) or ACG (antisense) core. [2)] HRE 4+5 are found at the same location, but on opposite strands

Table S2. Primer used to amplify human DEPP promoter

Forward (5′-3′)	Reverse (5′-3′)	Lenght
[1)] acgg <u>GGTACC</u> GTTGAGGAGCCCCTCGATGATCA	[1)] acgcc <u>GCTAGC</u> CCTGAGCAGTGTGAAGAACCTTC	3759 bp

[1)] Extra nucleotides shown in small letters, cloning restriction sites underlined.

Table S3. Sequence of the 3759 kb regulatory region (5′ → 3′)

GTTGAGGAGCCCCTCGATGATCAGAGTCCCTTCTTCCTGTGGGGGTCCACACATCAGCCCTTCCC
CCAGAGCAGCTGCCTACCTTCCCTGTGCCCCCTAGGGATGGGATGCTCTGAACACTCAGGCCTT
GCACATCCCTCCTCCTGAGTGGCCCCATCCCCTCAGGAAGGACCATCAGCCACTACGTGCCCTG
GGGCGTGTGACCAGGCCCTGTGGGAGGGAGCCTGAGATGTGCTCAGCTCATGCCTGGCCAGGG
GCACAGCGCAGAACACAGTGGCTGAGTCAGGTGTCCCACAGGAAGCCAGCTGCTGGCCGGCA
GGCACGTCCACACCGCGGTGAAGAAGTGGTTCCAGGGACGCACAGCCTGTCTGCTCATGCCAA
GCAGGGGACCTCTCTGCCCACACTGCACCTGGGAGGGGGTCCTGCGCCACCAGGTCCTATGGC
CACCAGGCCCGGCAGGGTCCCACACCCACCAGAGCAGAAGTGGGCACTCTCTGTCCTAGCTGT
GTCTCAGCCTCACCCTTCTCCCGGTGCCTTCCAACAGCAGCAGCGTCCACTTGCCAGGGCTGAC
AAAGGGTCTTGCTTTTCTCACCACTGCTTAGACATCACTAGCCCTATATCCCATACCAGAATTTC
TACAACAGCCTTCATCCCATGTCCCCAACCTCCCTCGGTTTCCCTAGGTCTGCAGGTGACATGGC
TTCTCTCCTGCTGCCAACCTGGGGCCCTAATCAACAGAGTCCTCTGCACGCACCTTGCTAGTAGT
GCTGGTTGGCACCCCTTGGGCCCTTGATTCAGTGCCTCTGACCTATTCCAGGCCTCTGTCTCTGC
GAGACCACCCCAGGCCTCTGTGCCAATCCCGGTCACTCTGTGTGTAAGCCTGGCCAAGCTGAGT
CATTTGCCAGAATACTTGCCAGCAACCTCGGCTGCCCTCTACCCAGAGCCGCAGTCTCGAAACT
ACACACTCCTAATGTGCTCTGACACTAGTCTTCCCACAATTAACTTTGAATATCATTCTCTCATT
GCCATCTCAACCAACATTTAATTTTATTATTTATGTTTTATTGTGATAAGGACATTTAACATAAG
AACTACCCTCTAAACAAACTGTTCAGTGCACAATACTGTATTGTTCACTGTAGGCACAACGTTG
TCCAGCCCATATCCAGGACTTACTCATCTTGCATAGCTGAAACATTATATCAGCTGCACAGCAA
CTCTCCACTTCCTCCTCCCCTGCCCCAGTCCCTGCAACCACCATTTTCACTCTCTGCCTCTATGAG
CCTATTTTAGATTCCTTATATAAGGGAATCATGCAGTATTTCTGACCCATATTTTAGATTCACAA
ATCCCATTTCTCTCTCCTCGTTGGCACTAAAAGCCCTGTGAGAACAGGGACGCCATGGAAACTT
CTTTGTATCCCTGGCAGCACTCAGGAAGACCCTGCACAATGTGGATTTCATGGTAACTTAGAAA
TGGGGTTTCTAGGTTCAAACATCCTGCCATCCACAGAGAAAATAGTGTATCCAAAGGTTATCAA
ATATACACACAGCTTCCAGATATGACCTTGCACTATCTGCTAAAAAGGAAAACAAACAAGCAG
AAACCAGAGCTGTTTTTGATACTCACTGGTGCTCTACAAGCACAGATGTGTCACTGAGCTTCCT
ATGAAGGAGAGGCCAGCATGGATCTATGGAGTTCAAAGGTCTATGGGGGGTTCTGTTCTTGCCT
CTTGGTCTCGGGTGCCTCTCAGTGGAGAGAGCTTGTGGGGATGGCTCACTCATGGAGACCCACT
GCCCTCCAGCCCCACCCTGGACGCCAGGCAGAGGAAGATGGAGCAGACACAGCCCTGGCCAC
GAGGGGCTCGGGCTTGGACACAGAAGGAGATGCAATGGAGCTTCTTGGAGGAGGTGCTTGTGC
ACTGCACCCTGGAAGACGAGTGAAGGGGGACGGGGAGGAAAGCTGAGCAGGTGGCCCTGACC
TCAGGACCTGCCTGGACAAGGTCGGGGGCTTGGCTGTACTGGGGACAGGCTGGCTCTCCATGA
CTCAGGCCAGACATGCCTACACAGGTGATGTGTGTGCCCCCAACATAGCCGCCCACTGTTCACC
CTCCCCCAGCTTGGCCGCATCTGAATGGAGGGAAAGCCCCGTCAGCCAGCAGCGAATTCTCCG
CCTCCCTCCCTCTTTTGGTCTCTTGACTAATCTGCTCATTGCTAATAAATTCAAAGAAGAGCCAG
CGAACTAGACAAAGGGTCTACAAAAGATATAAGAGCTTGGGCAAGGTGAGAAGTGCCATGTG
CCACTGAAATCCTGGTCACTGTCTCAGGCACTCCCTGCCCCGATGCCTTTGTGCACATGGAGTTC
CCACCTAGAATTCAAGAACTTGGGACTGCCGGTCCCTCTTGCTTGCCTAGGGAGTCTGGGGTCT
GCCCAGTACTCAGAGCTCAGGCTTGGGCTAACTCTCAAGACTCCTCTTTCTCCCCCACCTGCAG
CACAGGCAGGCGTGTTCCAGGTGGGGCTGTGGCTGGGGCAGAGGATCTGTGGCCTGTCTGGGG
AGTGCAGGAGCTTCCCAGGGGCTGCCTCTCTCATCCTTGGCAGCCTCCTTTGCTGGCATGGTCAC
TCCTGGACAGATGCAGTGGCTTTCTAATGTTGCCCAGGTGCTCCTTGGCAGCCCCCCAGCCCCA
GCCCCAGCCCAGTCCCAGCAGCTGCAGCGATCCTTTGAAGCAGAAGCCAGACTGCAGTGCCCT
TGTTCAAACCCTGCAGTCTTCCCCTCCCTCCTAGAGGGAAACACAGCATCCACCCCAAGGTCCA
CTGACCCCCTCCTACATGGGCCAGTCTGCAGGCCCCTTGCTGTGCCCTGAGCATGTCTGTCCGGC
GCGCCCTCACCACAGGGGCTTTGCACGTGCTGCCCCTGCCACCTGGAATGTGCATTTTCCCATTT
TTTTCAGGTTTCTGCTCCAATATCTGCTCTTAGGGAAGACACCCCTGACCACTGTACCTGTGGCG
GCAGCCCACCACAGCATGCCACTGCGCTGCTTTCCTTCAGGCAGTTCTTGCTGTTGCCTGAATCA
CAGCACCCATTCATCTCTGGCTTGATCTCCTCCCTTACTAGAATATGAGCTCCAGGGGGCCACA
CCTTTGTCCGCTGCTACGTTTGCTGTGCTTAGTGCATTCACAGCACACACTAGCACCTCATATCT
GGTGAACAAGTAAACAAACAAATCCTCCGAAAGCACCCCATCTGGGGTGTAAGAAGACCAGG
GTCAGACCCCGCAGACCGCCCTTGGTCCCTTGCGAGCAGGGTGTGTCTCTCCTAGGAGCAGGCC

TAGGGACCCAGCCTCAGCTGCCTCCAGACTGGGGTCCTGTGCTGCTGATTTTTCATCAAAGTTGC
CAAGGACCTGGCCCAACTTCACAGGAGTCATGATGGGGTGGTGTAACACAGAGTGGGAAACA
AGCTTTGGGGCAGGGGTGGCCCTGGGGCTACCTGTGAGGGCCCAGTGGAACCCCTCCCTCTCCC
AGTGCCCCTACTTGACCAGCAGTGAACCCACCCCTTTCCTGCCCTGCAGCGTAAACATTCCCCA
GCCTCCTACAGGCAGAAAAACACCTCCAAGCTGGCAGCTGGGCCAGGCCCCGCCCACCAAGCT
GTTTTCCTAAGACAGCCTTCCGAGTAGGCTATTTTTGGCCCTCGTGACAGTGATTGATAGCTGCT
GGGAAGGTATAAAAGCAGCTTGCCTGCGAAGGTTCTTCA**C**[1)]ACTGCTCAGG

[1)] TSS is bold and underlined. TSS is base 0 and the first base after the TSS is +1

Table S4. Primers used to generate BLOCKs

BLOCK		Forward (5′-3′)	Reverse (5′-3′)	Lenght
BLOCK 1 [1)]		acgg GGTACC AGCTCAGGCTTGGGCTAACT	acgcc GCTAGC CCTGAGCAGTGTGAAGAACCTTC	1318
BLOCK 2 [1)]		acgg GGTACC TCTGACACTAGTCTTCC	acgcc GCTAGC AGTTAGCCCAAGCCT	1485
BLOCK 3 [1)]		acgg GGTACC GTTGAGGAGCCCCTCGATGATCA	acgcc GCTAGC TGTGGGAAGACTAGTGTCAGAGC	997
BLOCK 1 + 2 [1)]		acgg GGTACC TCTGACACTAGTCTTCC	acgcc GCTAGC CCTGAGCAGTGTGAAGAACCTTC	2783
BLOCK 2 + 3 [1)]		acgg GGTACC GTTGAGGAGCCCCTCGATGATCA	acgcc GCTAGC AGTTAGCCCAAGCCT	2461
BLOCK 1 + 3 [1)]	Step 1	acgg GAGCTC AGCTCAGGCTTGGGCTAACT	acgcc GCTAGC CCTGAGCAGTGTGAAGAACCTTC	1318
	Step 2	acgg GGTACC GTTGAGGAGCCCCTCGATGATCA	acgcc GAGCTC TGTGGGAAGACTAGTGTCAGAGC	997

[1)] Extra nucleotides shown in small letters, cloning restriction sites underlined.

Table S5. Primers used for 5′ end truncations

HREs in fragments	Forward (5′-3′)	Reverse (5′-3′)	Lenght
1 - 26 [1)]	acgg GGTACC GAGGGAGCCTGAGATGTGCT	acgcc GCTAGC CCTGAGCAGTGTGAAGAACCTTC	3543
1 - 25 [1)]	acgg GGTACC CCTGGCCAGGGGCACAGCGC	acgcc GCTAGC CCTGAGCAGTGTGAAGAACCTTC	3514
1 - 24 [1)]	acgg GGTACC TGAAGAAGTGGTTCCAGGGA	acgcc GCTAGC CCTGAGCAGTGTGAAGAACCTTC	3423
1 - 19 [1)]	acgg GGTACC TGCTAGTAGTGCTGGTTGGC	acgcc GCTAGC CCTGAGCAGTGTGAAGAACCTTC	3003

[1)] Extra nucleotides shown in small letters, cloning restriction sites underlined.

Table S6. Primers used for site directed mutagenesis

HRE		consensus	Forward (5′-3′)	Reverse (5′-3′)
29	wt	GGATG**<u>TGC</u>**	AGGAGGGATGcatAAGGCCTGAGTGT TCAGAG	CCTGAGTGGCCCCATCCC
	mut	GGATG**<u>CAT</u>**		
28	wt	CT**<u>ACG</u>**TGC	ATCAGCCACTgtaTGCCCTGGGGC	GGTCCTTCCTGAGGGGAT
	mut	CT**<u>GTA</u>**TGC		
27	wt	GGG**<u>CGT</u>**GT	TGCCCTGGGGtacGTGACCAGGC	CGTAGTGGCTGATGGTCC
	mut	GGG**<u>TAC</u>**GT		
26	wt	AGATG**<u>TGC</u>**	GCCTGAGATGcatTCAGCTCATGCC	TCCCTCCCACAGGGCCTG
	mut	AGATG**<u>CAT</u>**		
25	wt	GG**<u>ACGTGC</u>**	CGCGGTGTGGgtacatCTGCCGGCCA	GTGAAGAAGTGGTTCCAGG
	mut	GG**<u>GTACAT</u>**		
20	wt	GTGCG**<u>TGC</u>**	GCAAGGTGCGcatAGAGGACTCTG	TAGTAGTGCTGGTTGGCA
	mut	GTGCG**<u>CAT</u>**		
